# Prevalence of human papillomavirus genotypes and relative risk of cervical cancer in China: a systematic review and meta-analysis

**DOI:** 10.18632/oncotarget.24169

**Published:** 2018-01-11

**Authors:** Hui-Hui Xu, Kai Wang, Xing-Jun Feng, Shan-Shan Dong, Aifen Lin, Ling-Zhi Zheng, Wei-Hua Yan

**Affiliations:** ^1^ Laboratory of Gynecologic Oncology, Medical Research Center, Taizhou Hospital of Zhejiang Province, Wenzhou Medical University, Linhai, Zhejiang, China; ^2^ Department of Gynecology and Obstetrics, Taizhou Hospital of Zhejiang Province, Wenzhou Medical University, Linhai, Zhejiang, China; ^3^ Human Tissue Bank, Taizhou Hospital of Zhejiang Province, Wenzhou Medical University, Linhai, Zhejiang, China

**Keywords:** human papillomavirus, cervical cancer, cervical precancerous lesion, genotype, meta-analysis

## Abstract

High-risk HPV (hrHPV) is related to cervical carcinogenesis, although clinical data comparing the natural history and carcinogenic potential of type-specific HPV remain limited. Furthermore, the nationwide prevalence rates of overall and type-specific HPV among women with cervical precancerous lesions and cancer have not been reported. Here, a meta-analysis was performed for type-specific HPV distribution among 30,165 HPV-positive women, including 12,094 invasive cervical cancers (ICCs), 10,026 cervical intraepithelial neoplasia grade 2/3 (CIN2/3), 3246 CIN1, and 4799 normal cervices from 45 PCR-based studies. We found that HPV16 was the most common hrHPV type involved in cervical disease. The HPV16 positivity rate varied little across normal (22.7%) and CIN1 individuals (23.6%) but increased through the CIN2 (37.6%) and CIN3 patients (51.9%) to 65.6% in ICC cases. HPV16, 18, 35, 39, 45, and 59 were more frequent in ICC than CIN3, with ICC:CIN3 ratios ranging from 2.3 for HPV18 to 1.1 for HPV35/45. HPV31, 33, 52, and 58 were more frequent in CIN3 compared with normal cervices but less common in ICC compared with CIN3 (ICC:CIN3 ratios ranging from 0.6 for HPV58 and 0.4 for HPV52). The ICC:normal ratios were particularly high for HPV18, 52 and 58 in West China (4.1, 3.9 and 2.9, respectively) and for HPV45 and 59 in North China (1.6 and 1.1, respectively). In summary, this study is the most comprehensive analysis of type-specific HPV distribution in cervical carcinogenesis and could be valuable for HPV-based cervical cancer screening strategies and vaccination policies in China.

## INTRODUCTION

Cervical cancer is the second most common female malignancy worldwide. In China, approximately 98,900 new cases were reported in 2015, which accounted for 18.7% of the global incidence [1]. Human papillomavirus (HPV), commonly transmitted through sexual activity, was recognized as an important cause of cervical precancerous lesions or cancer. To date, 14 HPV types have been classified as “high-risk” for their strong carcinogenic potentials. HPV16 and HPV18 are well-known carcinogenic genotypes; additionally, HPV31, 33, 35, 39, 45, 51, 52, 56, 58, 59, 66, and 68 are also strongly associated with cervical cancer [2, 3]. In this scenario, numerous epidemiologic studies, most from Europe or the United States, have shown that nearly 100% of patients with cervical cancer test positive for HPV [3–6]. These results have led to approaches in which HPV testing is an option for primary screening with HPV16/18 genotyping along with a cocktail test of 12 other high-risk HPV (hrHPV) genotypes [7]. HPV testing is more sensitive than cytology in primary screening and offers a longer negative predictive value for cervical cancer [5–7].

Cervical cancer incidence and mortality have been increasing and are a major public health problem in China. China has the world's largest population (approximately 1.37 billion), with approximately 70% of individuals living in rural regions where the incidence of cervical cancer is extremely high [8]. Most cervical cancer screening in China is opportunistic, and cancer incidence rates vary throughout the country [8, 9]. In addition, economic factors, education, and HPV genotype distribution, which vary among geographical regions, must also be taken into account. Moreover, comprehensive clinical data on HPV genotype prevalence and relative risk of cervical cancer in Chinese females are lacking.

In this study, we performed a systematic review and meta-analysis of type-specific HPV distribution across the complete spectrum of cervical diagnoses from normal to invasive cervical cancer (ICC). With updated data and detailed analyses, we further evaluated how influential parameters (such as geographical region, HPV DNA source, and PCR primers) affected the results from the meta-analysis.

## RESULTS

### Summary of eligible studies

**Figure 1:**
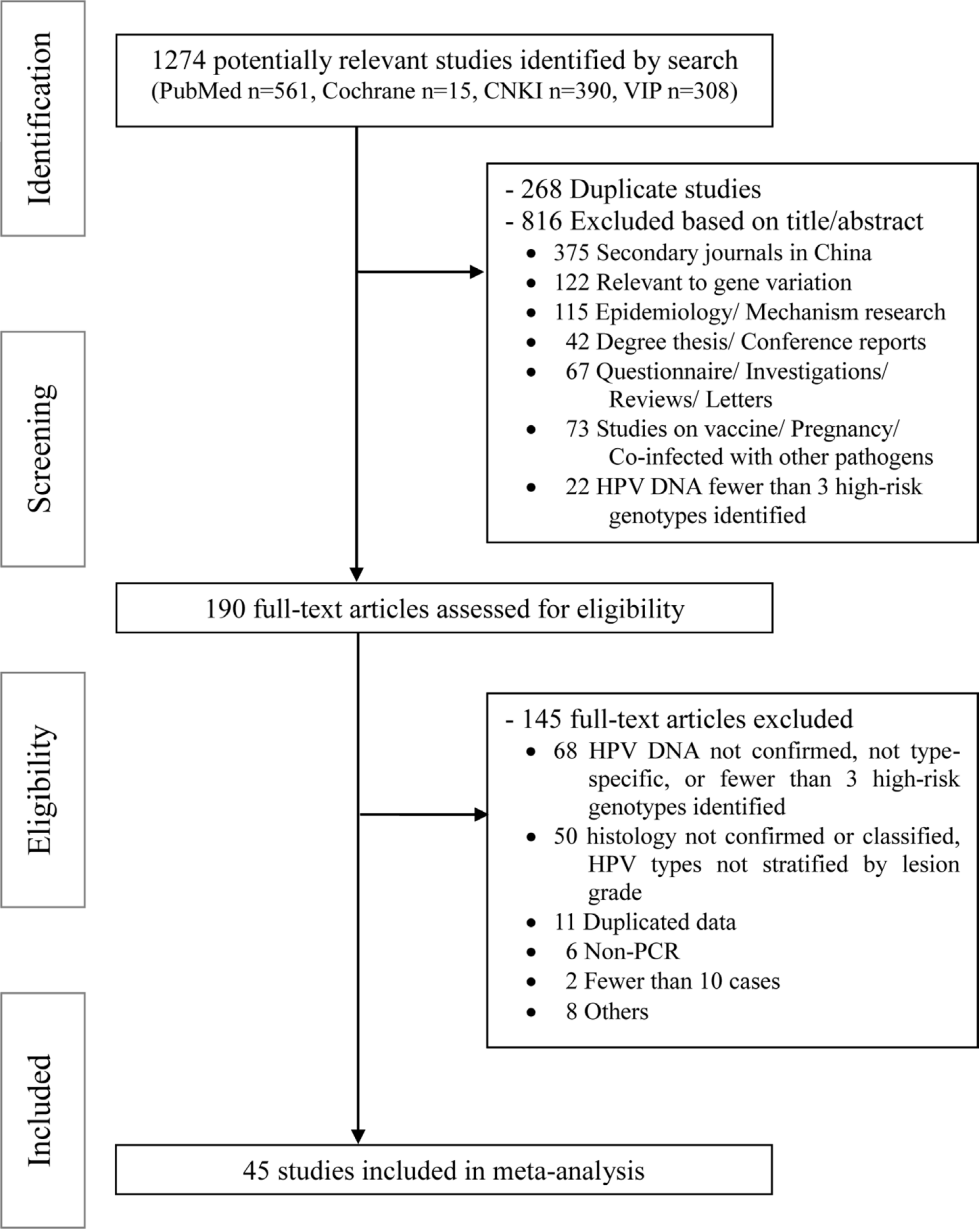
PRISMA flow diagram for identification of studies for meta-analysis

We retrieved 1274 citations from the search strategy (Appendix S1), and 190 potentially relevant articles were identified for full-text review. Data were abstracted from 45 eligible studies that met the eligibility criteria [10–54]. The study flow diagram is shown in Figure 1.

### Study characteristics

Forty-five eligible studies published between 1996 and 2016, including forty-one single-center [10–50] and four multi-center studies [51–54], were documented in this review. Among these studies, 12 were conducted in East China [10–21], 3 in South China [22–24], 4 in West China [25–28], 14 in North China [29–42], 3 in Taiwan [43–45], 4 in Hong Kong [46–49], and 1 in Macao [50].

**Figure 2:**
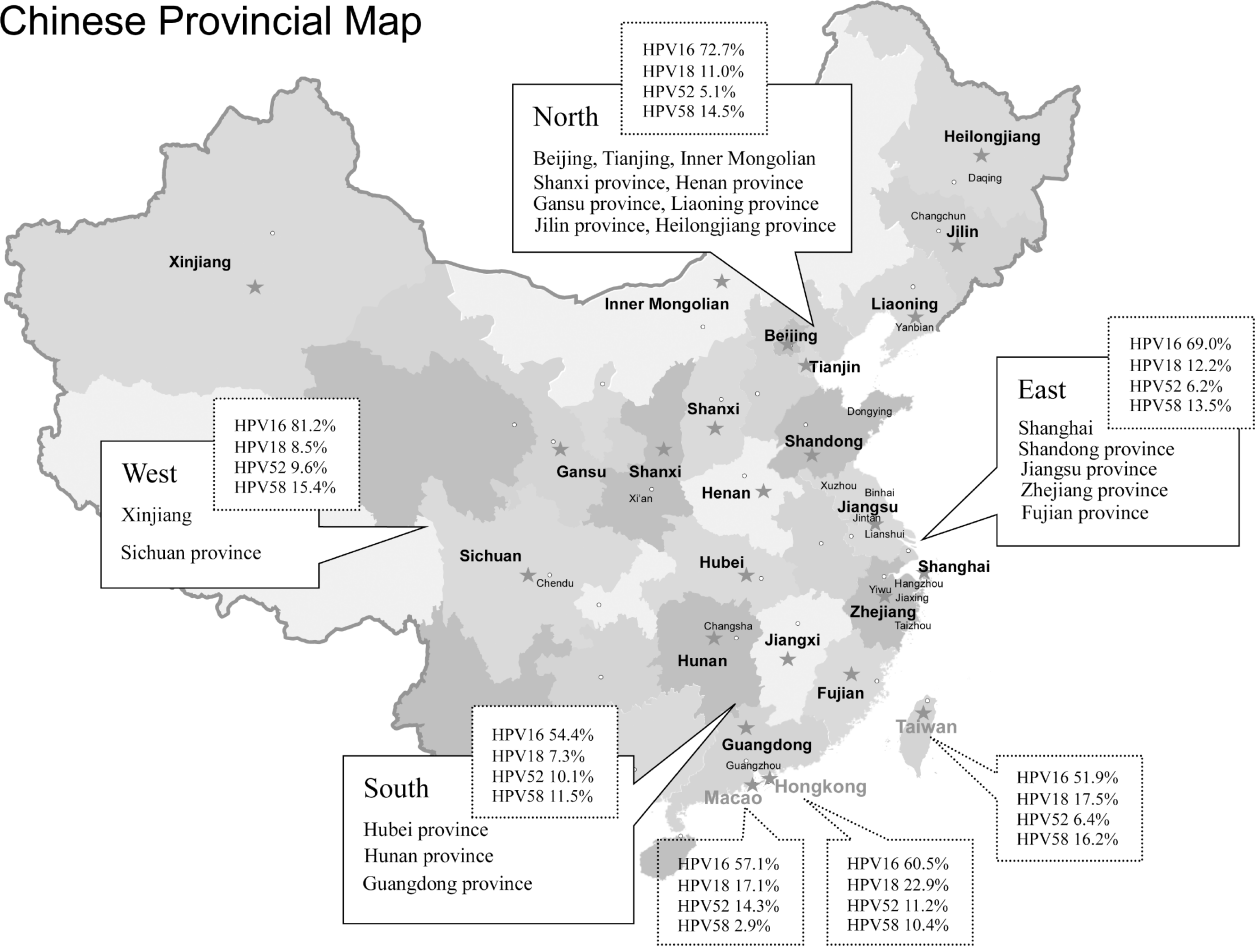
National map of China showing all the geographical sites included in this meta-analysis

A total of 49,997 eligible women from 32 provinces and municipalities were recruited to participate in these studies (Figure 2). Out of 49,997 women tested for overall HPV prevalence, 19,361 (38.7%) had normal cervices, 4877 (9.8%) CIN1, 11,967 (23.9%) CIN2/3, and 13,792 (27.6%) ICCs. The overall HPV distribution stratified by cervical disease grade and geographical region is shown in Table 1. Eastern China contributed the largest number of samples (20,292, 40.6%), followed by Northern China (13,390, 26.8%), Taiwan (6521, 13.0%), Hong Kong (3297, 6.6%), and Western China (2428, 4.9%). Of the 49,997 women, 1496 (3.0%) were non-Han Chinese women including 1431 Uyghur [28] and 65 Mongolian [38].

**Table 1:** Overall HPV prevalence in 49997 women from China, stratified by cervical disease grade and geographical region

	Total (studies = 45)		ICCs (studies = 39)		CIN2/3 (studies = 32)		CIN1 (studies = 25)		Cervicitis/Normal (studies = 21)	
	Tested, *N*	HPV positive, *n*	Tested, *N*	HPV, *n* (%)	Tested, *N*	HPV, *n* (%)	Tested, *N*	HPV, *n* (%)	Tested, *N*	HPV, *n* (%)
Eastern of China (studies = 12)	20292	10758	5043	4123 (81.8)	3190	2683 (84.1)	1928	1256 (65.1)	10131	2696 (26.6)
Southern of China (studies = 3)	1614	1255	1554	1205 (77.5)	60	50 (83.3)	0	-	0	-
Western of China (studies = 4)	2428	1462	334	293 (87.7)	866	759 (87.6)	249	168 (67.5)	979	242 (24.7)
Northern of China (studies =14)	13390	7329	1940	1803 (92.9)	3826	3057 (79.9)	1279	796 (62.2)	6345	1673 (26.4)
Taiwan (studies = 3)	6521	4332	2475	2412 (97.5)	1703	1421 (83.4)	542	322 (59.4)	1801	177 (9.8)
Hong Kong (studies = 4)	3297	2743	785	747 (95.2)	1622	1367 (84.3)	785	618 (78.7)	105	11 (10.5)
Macao (studies = 1)	99	94	36	35 (97.2)	63	59 (93.7)	0	-	0	-
Multi-center (studies = 4)	2356	2192	1625	1476 (90.8)	637	630 (98.9)	94	86 (91.5)	0	-
Overall (studies = 45)	49997	30165	13792	12094 (87.7)	11967	10026 (83.8)	4877	3246 (66.6)	19361	4799 (24.8)


Abbreviations: ICC: invasive cervical cancer; CIN: cervical intraepithelial neoplasia.

### Overall HPV prevalence and meta-analysis

**Figure 3:**
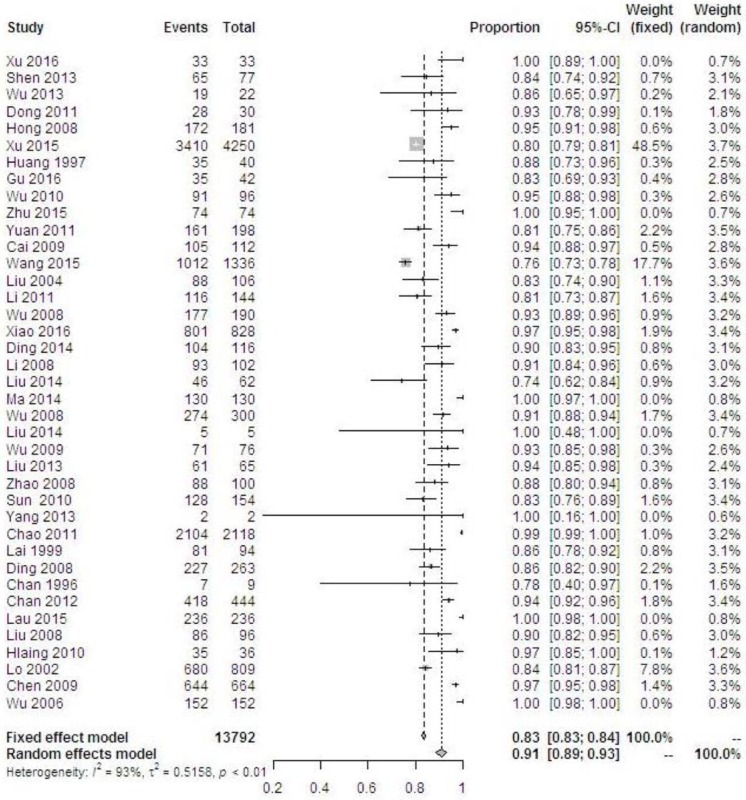
Forest plot of the overall HPV prevalence in cervical cancer

A total of 30,165 women were HPV-positive. Overall HPV prevalence increased with the degree of cervical disease severity from 24.8% in normal cervices to 87.7% in ICC (*P* < 0.001) (Table 1). The pooled prevalence of overall HPV types among women with ICC was 91.1% (95% CI 88.7–93.1%) and displayed significant heterogeneity, I^2^ = 93.2%, *P* < 0.0001 (Figure 3). Differences in the HPV positivity rate by geographical region varied obviously among pathological categories. In normal cervices, the overall HPV prevalence varied substantially by region, ranging from approximately 10% in Taiwan/Hong Kong to more than 20% in mainland China. For ICC, the overall HPV prevalence was consistent (more than 95%) in Taiwan, Hong Kong, and Macao but ranged from 77.5% to 92.9% and yielded an average of 83.7% (95% CI 82.9–88.2%) in mainland China. The overall HPV prevalence rates in SCC, ADC and unspecified ICC were 86.9% (5840/6721), 71.5% (459/642) and 90.1% (5795/6429), respectively.

After stratified by HPV DNA source, we found that the prevalence rates of overall HPV types in cervical cancer from tissues were consistently significantly higher than that from exfoliated cells in all geographical regions (*P* < 0.05). With regard to PCR primers, HPV prevalence was higher in samples that were tested using SPF1/GP6+ (99.3%, 95% CI 99.0–99.7%) and/or SP10 (95.1%, 95% CI 93.6–96.7%) primers when compared with MY09/11 (86.1%, 95% CI 84.9–87.3%) and/or PGMY09/11 (83.6%, 95% CI 82.6–84.5%) primers (*P* < 0.001). Meanwhile, the meta-analysis of the HPV prevalence in women with cervical cancer based on HPV DNA source, different region, publication calendar period, and PCR primers is shown in Table 2.

**Table 2:** Meta-analysis of the HPV prevalence in women with ICC, stratified by HPV DNA source, across region, publication calendar period, and PCR primers

	Total				Cells-based detection				Tissue-based detection			
	No. of studies	No. of cases	*P* for heterogeneity	Pooled prevalence % (95% CI)	No. of studies	No. of cases	*P* for heterogeneity	Pooled prevalence % (95% CI)	No. of studies	No. of cases	*P* for heterogeneity	Pooled prevalence % (95% CI)
**Total**	**39**	**12094**	**<0.0001**	**91.1 (88.7–93.1)**	**24**	**7557**	**<0.0001**	**89.6 (86.4–92.0)**	**15**	**4537**	**<0.0001**	**92.9 (87.9–95.9)**
**Region**												
Mainland	28	7424	<0.0001	89.1 (86.3–91.4)	20	6753	<0.0001	89.1 (85.7–91.7)	8	671	0.0008	89.2 (83.9–92.9)
Taiwan	3	2412	<0.0001	94.7 (70.6–99.3)	1	227	NA	NA	2	2185	<0.0001	96.8 (57.6–99.9)
Hong Kong	4	747	0.0081	92.9 (83.9–97.1)	2	425	0.0652	90.1 (68.3–97.5)	2	322	0.0059	98.1 (50.5–99.9)
Macao	1	35	NA	NA	0	0	NA	NA	1	35	NA	NA
Multi-center	3	1476	<0.0001	95.9 (81.8–99.2)	1	152	NA	NA	2	1324	<0.0001	96.8 (57.6–99.9)
**Publication calendar period**												
1996-1999	3	123	0.7527	85.9 (79.1–90.7)	1	7	NA	NA	2	116	0.8364	86.6 (79.7–91.4)
2000-2009	13	2857	<0.0001	91.6 (88.2–94.1)	5	918	0.0035	92.0 (87.1–95.2)	8	1939	<0.0001	91.2 (85.8–94.7)
2010-2016	23	9114	<0.0001	91.4 (87.8–94.0)	18	6632	<0.0001	88.7 (84.9–91.7)	5	2482	<0.0001	97.3 (81.3–99.7)
**PCR primers**												
MY09/11	15	2741	<0.0001	90.3 (84.2–94.2)	9	2167	<0.0001	91.2 (79.9–96.4)	6	574	0.0627	89.0 (84.7–92.2)
PGMY09/11	14	5221	<0.0001	88.7 (84.6–91.8)	12	4899	<0.0001	87.8 (83.4–91.1)	2	322	0.0059	98.1 (50.5–99.9)
SP10	3	725	<0.0001	93.2 (66.4–98.9)	0	0	NA	NA	3	725	<0.0001	93.2 (66.4–98.9)
SPF1/GP6+	1	2104	NA	NA	0	0	NA	NA	1	2104	NA	NA
MY09/11+GP5+/6+	6	1303	0.0003	90.4 (84.8–94.1)	3	491	0.0092	91.8 (80.6–96.8)	3	812	0.0167	90.3 (80.5–95.5)

95% CI: 95% confidence interval; NA: not available.

### Type-specific HPV prevalence and risk of cervical cancer

Type-specific HPV prevalence in HPV-positive women stratified by cervical disease grade is shown in Supplementary Table 3. HPV16 was the most frequently detected hrHPV type in every grade. HPV16 positivity rate varied little between cervicitis and normal tissues (22.7%, 95% CI 21.5–24.0%) and CIN1 (23.6%, 95% CI 22.0–25.3%) but increased through CIN2 (37.6%, 95% CI 35.5–39.7%) and CIN3 (51.9%, 95% CI 50.1–53.7%) to reach 65.6% (95% CI 64.7–66.4%) in ICC. HPV18 positivity varied very little between cervicitis and normal tissues and CIN3 (5.6–7.9%) but increased to 12.6% (95% CI 12.0–13.2%) in ICC. HPV58 was the second most common type in ICC (12.6%, 95% CI 12.0–13.2%). Notably, HPV52 was the second most common type in cervicitis/CIN1/CIN2 (16.3–22.0%), but the prevalence rate decreased remarkably through CIN3 (15.7%, ranked third) and reduce to 6.5% in ICC (ranked fourth). For the next five most common hrHPV types in ICC, including HPV33, 31, 59, 45, and 39, the positivity rate ranged from 2.1% to 5.5%. For the next five least common hrHPV types in ICC, including HPV51, 56, 68, 35, and 66, the positivity rate ranged from 0.7% to 1.4%. HPV16, 18 and 45 were the only hrHPV types found more frequently in ICC than in cervicitis/normal samples, with the ICC:cervicitis/normal ratios of 2.9, 2.2 and 1.4, respectively. HPV31, 33, 52, and 58 were more frequent in CIN3 in comparison with cervicitis/normal samples, but less common in ICC compared with CIN3 (ICC:CIN3 ratios ranging from 0.6 for HPV58 and down to 0.4 for HPV52). HPV16, 18, 35, 39, 45, and 59 were more frequent in ICC than CIN3, with ICC:CIN3 ratios ranging from 2.3 for HPV18 to 1.1 for HPV35/45.

**Figure 4:**
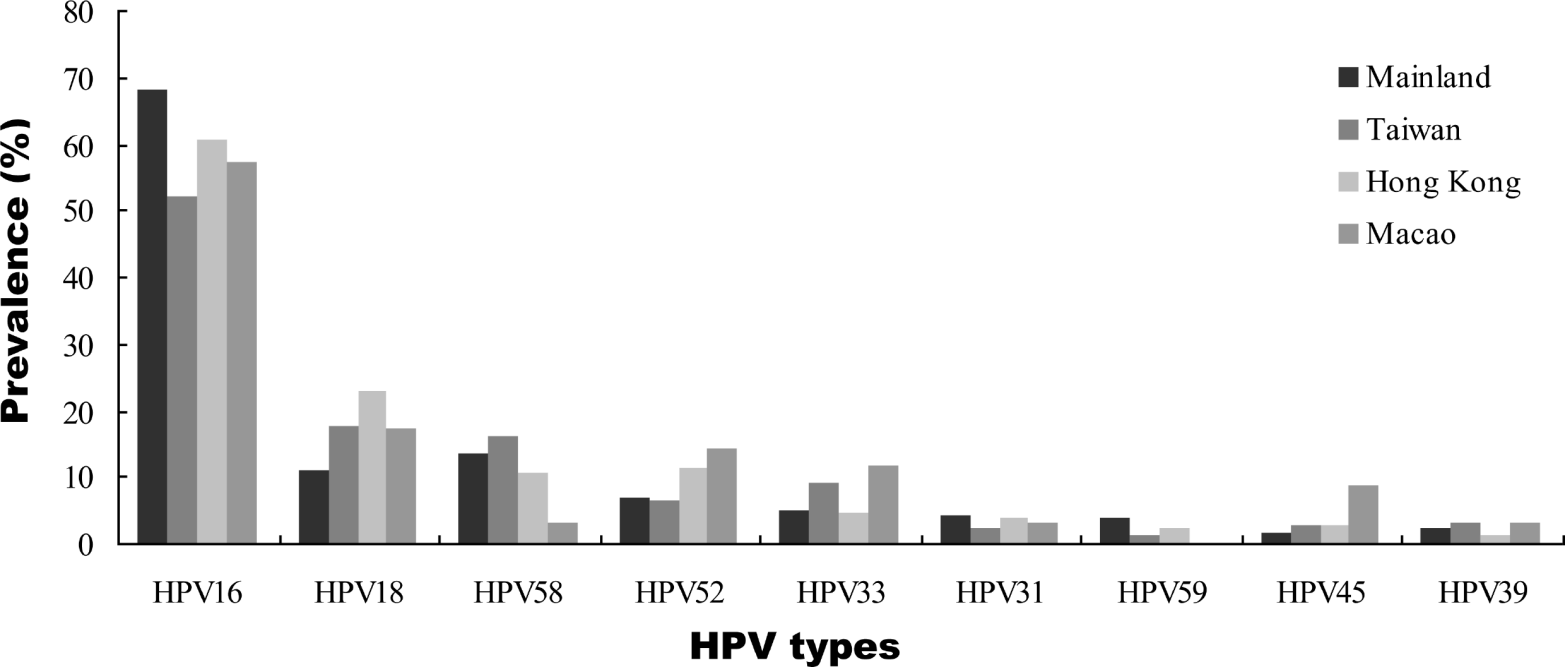
Type-specific HPV distribution by geographic region in Chinese women with ICC

When stratified by geographical region, we found increased HPV16 positivity with lesion severity to be similar in all regions, with ICC:cervicitis/normal ratios ranging from 2.4 in North China to 4.2 in East China (Figure 4 and Supplementary Table 1). The increased HPV18 positivity between normal and ICC cases was observed across all regions, with ICC:cervicitis/normal ratios between 1.8 and 4.1. For HPV58 and HPV52, a relatively elevated in ICC:cervicitis/normal ratio (2.9 and 3.9, respectively) was observed in West China and not apparent in other regions. With regard to HPV DNA source, we found that the prevalence of type-specific HPV in HPV-positive women with cervical cancer based on exfoliated cells were universally higher than that in tissues. HPV58 was the second most common type in exfoliated cells but ranked third in tissues (Table 3).

### Study quality and publication bias

The quality assessment of all eligible studies was based on the Agency for Healthcare Research and Quality (AHRQ) scale, which is shown in Supplementary Table 2, and the assessment results provided reasonable confidence in the reliability of the meta-analysis. The methodological quality assessment was considered high in thirty-four studies [10–15, 17, 19–23, 25–32, 39–45, 47–53] and moderate in eleven studies [16, 18, 24, 33–38, 46, 54]. Five studies had full scores for the representativeness of the cross-sectional study [10, 17, 20, 23, 43], but most of them lacked scores because subjects were not consecutive. Other studies lacked scores for incomplete follow-up data. Egger's and Begg's tests were performed to assess the publication bias and proved to be insignificant (both *P* = 0.33).

## DISCUSSION

HPV is a sexually transmitted infection, and high-risk HPV types have been shown to be the etiological agents of cervical cancer. Two meta-analyses have been published on the distribution of HPV types in the cervix among Chinese females, but the results were controversial [55, 56]. First, selected samples were identified by cytology or histology for study inclusion criteria. It is worth noting that the distribution of HPV types in cytology findings is different from those in histology assessment, which is the gold standard for diagnosis. Second, HPV testing might be especially useful for the detection of ADC, which can be difficult to identify using cytology [57]. Our study is the first to analyze nationwide clinical data on HPV types across the complete spectrum of cervical disease confirmed by cervical pathological diagnosis. The objective was to compare the correlation between type-specific HPV infections and the risk of cervical cancer. With the updated data and more detailed analysis, in this study, geographical region, HPV DNA source, and PCR primers, which have not been included in previous studies, were further analyzed.

In these summarized data from China, we found that HPV16 was the most frequently detected hrHPV type in each cervical disease grade, and the positivity rate steadily increased from normal histology to ICCs among all geographical regions (Supplementary Table 1 and Supplementary Table 3), indicating an increased long-term risk for cervical cancer with HPV16 infection [58–60]. The same pattern was found for HPV18. The pooled prevalence rates of HPV16 and 18 in cervical cancer were 66.8% (95% CI 62.5–70.8%) (Supplementary Figure 1) and 11.8% (95% CI 9.8–14.1%) (Supplementary Figure 2), respectively. Our data showed that the HPV16 positivity rate was similar to the previously published global data (64.7 ± 3.6%) but lower for HPV18 (16.5 ± 2.9%) [58]. Compared with the rest of the world, the HPV16/18 prevalence for ICCs (78.2%) in this study was similar to that reported for Eastern Asia (77.5%), lower than Western/Central Asia (88.1%), Europe (83.1%), and North America (80.8%), and higher than Latin America (66.4%) [58, 61]. These results strengthen the hypothesis that the distribution of HPV16/18 variants is related to geographical or racial distribution [62], and HPV variants that differ in their biological properties may present differences in carcinogenic potential.

Consistent with previous epidemiological studies, HPV16, 52 and 58 were the dominant hrHPV types in the general Chinese population [63, 64]. Our analysis showed that HPV52 and 58 were the 2nd and 3rd most common hrHPV types among women with lower grade lesions. The HPV52 positivity rate decreased remarkably from normal histology (16.3%, ranked second) to ICC (6.5%, ranked fourth), but HPV58 was still the common type in ICC (12.6%, ranked second). These findings showed that HPV52 and 58 were important etiological agents for cervical carcinogenesis in China; however, HPV52 was less carcinogenic than HPV58. In addition, HPV52 and 58 were more frequent in CIN3 than in normal cervices, but less common in ICCs. This pattern was also observed for HPV31 and HPV33. These results suggested that HPV31, 33, 52, and 58 could confer higher risks for CIN3 than other non-HPV16/18 types, supporting the results from previous studies [58–60, 65].

Alpha-7 species containing HPV18, 39, 45, 59 and 68, which are proportionally overrepresented in ADC [66], are known to be less efficiently detected by routine cytological screening [67, 68]. In the present meta-analysis, a total of 5480 SCC and 459 ADC cases with positive HPV status were included (Supplementary Table 4). ADC constituted less than 8% of all ICCs; therefore, the type-specific HPV prevalence in ICC is mainly driven by the findings for SCC. Our results confirmed that alpha-7 HPV types are significantly higher in ADC (46.9%) than in SCC (10.1%) (*P* < 0.001). Moreover, HPV18 was the most common alpha-7 type (44.4% in ADC vs. 9.1% in SCC), and similar patterns were also found for HPV39, 45, 59 and 68.

HPV35, 51, 56, 66, and 68 types found in < 1.5% of ICC represented an important proportion of low-grade cervical lesions. The lower ICC:normal ratios suggested that such types have relatively low carcinogenic potential. Of note, the ICC:normal ratios for HPV45 and HPV58 were consistently higher, suggesting a higher carcinogenic potential compared with all other non-HPV16/18 types.

Meta-analysis on the prevalence of overall HPV types showed a higher rate detected in a subgroup of tissues than in exfoliated cells (Table 2). However, the prevalence of type-specific HPV in HPV-positive women with cervical cancer based on cells was universally higher than that on tissues (Table 3). Our findings suggested that the HPV prevalence in cells did not correspond well to that observed in tissues. It should be noted that the complex link between historical infections and current disease status might be related to HPV genome integration status [69]. In addition, HPV type-specific primers are usually designed to amplify shorter sequences of HPV DNA and might be more sensitive for detection of HPV DNA sequences. Indeed, our study showed that the HPV detection rate of ICC in Taiwan was much higher using SPF1/GP6+ (99.3%, 95% CI 99.0–99.7%) to amplify an 184-bp fragment when compared with other PCR primer sets.

It is necessary to consider the limitations of our meta-analysis while interpreting the results. First, potential heterogeneity associated with the cross-sectional study design and its inherent risk of bias, including the variations in population characteristics, PCR-based HPV detection protocols, quality of diagnosis and cervical screening strategies among the studies, must be considered. Second, heterogeneity could not be ruled out even given the pre-designed subgroup analysis by geographical region, PCR primer sets and publication calendar period. To address this issue, we chose the random effect model meta-analysis to combine data. Third, 77.4% of the patients included in the meta-analysis came from only two macro-geographical regions of mainland China (40.6% in the East and 26.8% in the North, respectively). Thus, one should be cautious when extrapolating our summary results to all regions of China. Fourth, several studies did not include for a broad range for type-specific HPV [15, 38–40, 46, 51, 53].

Our present meta-analysis suggested a significantly increased risk of cervical cancer associated with high-risk HPV genotype infection. HPV16, 18 and 58 were the most frequently observed genotypes in cervical cancer specimens and showed a strong association with the development of cancer. However, the association between type-specific HPV distribution and cervical cancer risk was slightly influenced by factors such as geographical regions, HPV DNA sources, and PCR primers. It should be noted that the laboratory standardization and quality assurance of HPV genotyping methods may increase data comparability and improve virological surveillance in the future vaccine era of China.

## MATERIALS AND METHODS

### Consent statement

As this study was a systematic review and meta-analysis, we did not include any humans and/or animals. This study was approved by the Institutional Medical Ethics Review Board of Taizhou Hospital in Zhejiang Province.

### Search strategy

This meta-analysis was performed in adherence with the guidelines outlined in the Preferred Reporting Items for Systematic Reviews and Meta-Analyses (PRISMA) [70] (Supplementary Table 5, PRISMA Checklist). We used PubMed/MEDLINE (NCBI), the Cochrane Central Register of Controlled Trials (Wiley), China National Knowledge Infrastructure (CNKI), and the VIP database for Chinese Technical Periodicals (VIP) to search for relevant articles published from the earliest date available to November 15, 2016. The search strategy is described in Appendix S1. Furthermore, we reviewed the references cited in the retrieved articles to search for additional relevant studies.

### Eligibility criteria

The participants were females from China who were included in studies of cervical disease associated with HPV. Eligible studies met the following inclusion criteria: 1) using consensus PCR primers, 2) overall and type-specific HPV elicitation, and 3) confirmed by cervical pathological diagnosis. The exclusion criteria were as follows: 1) duplicated data, 2) reviews, letters, conference reports or degree thesis, 3) studies on HIV patients or other sexually transmitted pathogens, 4) studies on gestational or vaccinated women, 5) HPV genotypes not stratified by lesion grade or fewer than 10 cases, and 6) HPV DNA types fewer than 3 high-risk genotypes identified.

### Data extraction

Two investigators (SS. Dong and XJ. Feng) independently extracted data from eligible studies. Disagreements were resolved by discussion or involvement of a third investigator (HH. Xu). For each eligible study, the following items were extracted: first author, publication year, region of China, study design, HPV DNA source, PCR primers, pathological diagnosis, sample size, and the number of overall or type-specific HPV-positive samples. For a subset of studies reporting such data, the overall prevalence of multiple infections (which may also include low-risk HPV types) was also extracted. Detail information on all included studies is presented in Supplementary Table 4. Each study was classified according to the following criteria: 1) four macro-geographical regions of mainland China (East, West, South, and North, respectively), Taiwan, Hong Kong, and Macao, 2) HPV DNA source (exfoliated cells, fresh biopsies, fixed biopsies), and 3) histological diagnosis (CIN1, CIN2, CIN3, ICC, squamous cell carcinoma (SCC), or adeno/adenosquamous carcinoma (ADC)).

### Quality assessment

Two investigators (LZ. Zheng and A. Lin) independently assessed the quality/risk of bias for each eligible study according the quality assessment forms from the AHRQ (https://www.ncbi.nlm.nih.gov/books/NBK35156/) [71]. A third investigator (K. Wang) resolved the disagreements. In this meta-analysis, the methodological quality of all eligible studies was assessed by AHRQ and included an 11-item yes/no/unclear response option: the “Yes” was scored as “1”, and “No” or “Unclear” was scored “0”. Study quality was assessed as follows: high quality = 8–11; moderate quality = 4–7; low quality = 0–3.

### Statistical analysis

We calculated the prevalence of the overall HPV and type-specific HPV (14 high-risk HPV types: HPV 16, 18, 31, 33, 35, 39, 45, 51, 52, 56, 58, 59, 66 and 68; 2 low-risk HPV types: HPV 6 and 11) among Chinese women with cervical precancerous lesions and ICCs. Type-specific HPV prevalence was defined as the proportion of HPV-positive women in which a particular HPV type was detected, so sample sizes differed among the type-specific analyses. Odds ratios (ORs) and relative 95% confidence intervals (95% CI) were calculated using SPSS version 15.0 (SPSS Inc., Chicago, IL). *P* values were two-sided, and statistical significance was accepted if the *P* value was 0.05 or less.

Meta-analysis was conducted using a random effects model. The I^2^ statistic quantified the heterogeneity among the studies, and *P* < 0.10 was considered indicative of significant heterogeneity. Forest plots were used to display the results graphically. To examine the potential publication bias, we used the Egger's and Begg's tests, where *P* < 0.05 was considered to be statistically significant. All analyses were performed using the R statistical software, and the Metaprop command was used as it provides appropriate methods for dealing with proportions with 100% [72].

**Table 3:** Type-specific HPV prevalence in HPV-positive women with cervical cancer, stratified by HPV DNA source

	Total			Cells-based detection			Tissue-based detection		
	*N*	*n*	% (95% CI)	*N*	*n*	% (95% CI)	*N*	*n*	% (95% CI)
**Type-specific HPV**									
HPV16	12020	7882	65.6 (64.7–66.4)	7483	5021	67.1 (66.0–68.2)	4537	2861	63.1 (61.7–64.5)
HPV18	12020	1516	12.6 (12.0–13.2)	7483	841	11.2 (10.5–12.0)	4537	675	14.9 (13.8–15.9)
HPV58	11984	1507	12.6 (12.0–13.2)	7476	1005	13.4 (12.7–14.2)	4508	502	11.1 (10.2–12.1)
HPV52	11744	759	6.5 (6.0–6.9)	7324	488	6.7 (6.1–7.2)	4420	271	6.1 (5.4–6.8)
HPV33	11099	608	5.5 (5.1–5.9)	7483	376	5.0 (4.5–5.5)	3616	232	6.4 (5.6–7.2)
HPV31	11181	393	3.5 (3.2–3.9)	7476	291	3.9 (3.5–4.3)	3705	102	2.8 (2.2–3.3)
HPV59	7178	186	2.6 (2.2–3.0)	3640	115	3.2 (2.6–3.7)	3538	71	2.0 (1.5–2.5)
HPV45	5659	118	2.1 (1.7–2.5)	2902	35	1.2 (0.8–1.6)	2757	83	3.0 (2.4–3.6)
HPV39	7337	162	2.2 (1.9–2.5)	4066	83	2.0 (1.6–2.5)	3271	79	2.4 (1.9–2.9)
HPV51	4931	69	1.4 (1.1–1.7)	2336	41	1.8 (1.2–2.3)	2595	28	1.1 (0.7–1.5)
HPV56	5648	74	1.3 (1.0–1.6)	3141	51	1.6 (1.2–2.1)	2507	23	0.9 (0.5–1.3)
HPV68	3582	48	1.3 (1.0–1.7)	3035	42	1.4 (1.0–1.8)	547	6	1.1 (0.2–2.0)
HPV35	5131	60	1.2 (0.9–1.5)	2456	32	1.3 (0.9–1.8)	2675	28	1.0 (0.7–1.4)
HPV66	2338	16	0.7 (0.4–1.0)	1874	16	0.9 (0.4–1.3)	464	0	NA

95% CI: 95% confidence interval; NA: not available.

## SUPPLEMENTARY MATERIALS FIGURES AND TABLES












