# Supplementary material for: Prevalence of human papillomavirus genotypes and relative risk of cervical cancer in China: a systematic review and meta-analysis
Source: Oncotarget. 2018 Jan 11;9(20):15386–97. doi: 10.18632/oncotarget.24169 (PMC5880612; doi:10.18632/oncotarget.24169)
Supplement: Supplementary file 1 [file oncotarget-09-15386-s001.pdf]

Prevalence of human papillomavirus genotypes and relative risk of cervical cancer in China: a systematic review and meta-analysis

SUPPLEMENTARY MATERIALS

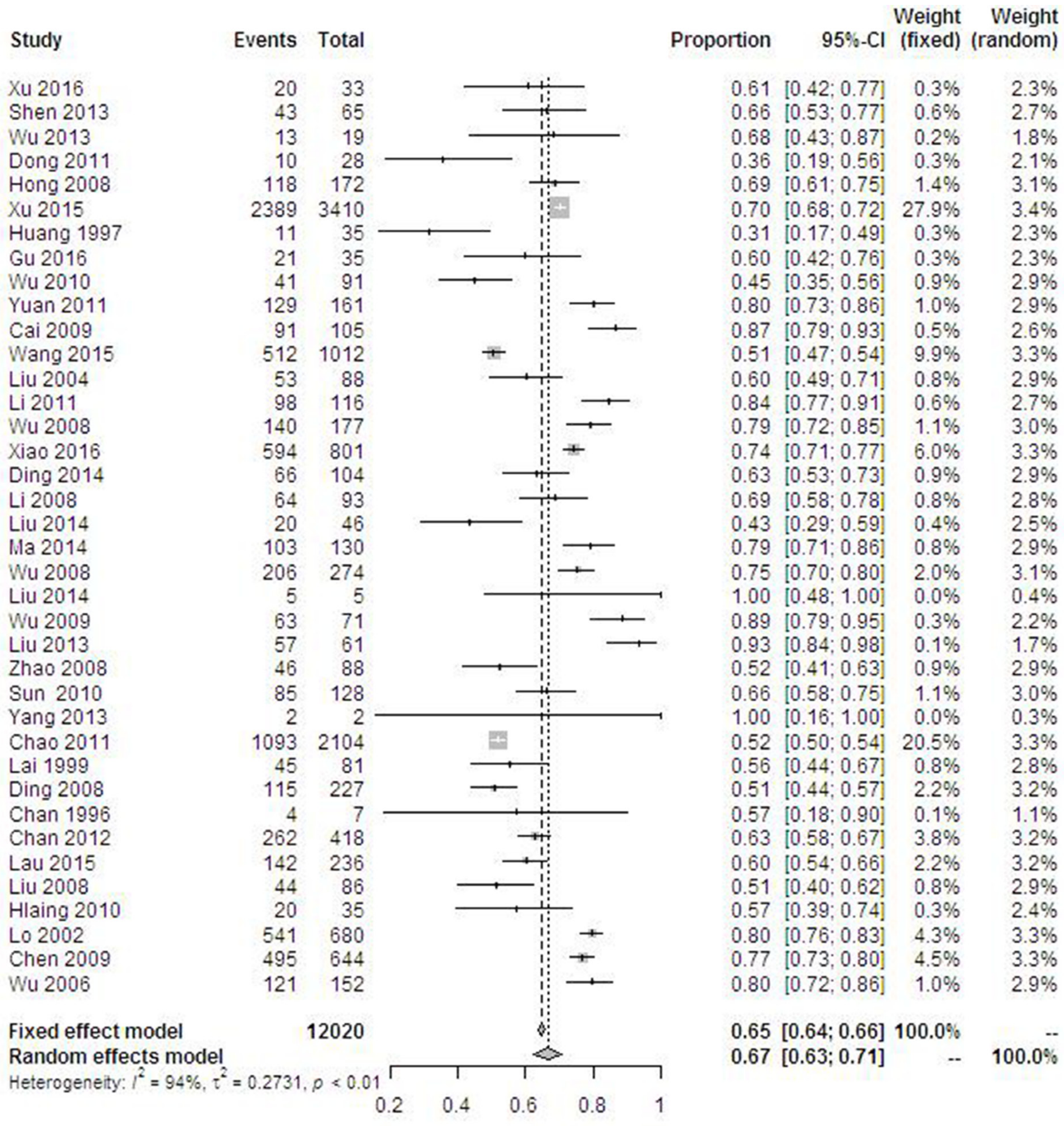

Supplementary Figure 1: Forest plot of the overall HPV16 prevalence in cervical cancer.

Study	Events	Total	Proportion	95%-CI	Weight (fixed)	Weight (random)
Xu 2016	3	33	0.09	[0.02; 0.24]	0.2%	1.7%
Shen 2013	4	65	0.06	[0.02; 0.15]	0.3%	2.0%
Wu 2013	1	19	0.05	[0.00; 0.26]	0.1%	0.8%
Dong 2011	5	28	0.18	[0.06; 0.37]	0.3%	2.1%
Hong 2008	17	172	0.10	[0.06; 0.15]	1.2%	3.2%
Xu 2015	424	3410	0.12	[0.11; 0.14]	29.0%	3.9%
Huang 1997	12	35	0.34	[0.19; 0.52]	0.6%	2.7%
Gu 2016	0	35	0.00	[0.00; 0.10]	0.0%	0.5%
Wu 2010	20	91	0.22	[0.14; 0.32]	1.2%	3.2%
Yuan 2011	8	161	0.05	[0.02; 0.10]	0.6%	2.7%
Cai 2009	4	105	0.04	[0.01; 0.09]	0.3%	2.1%
Wang 2015	74	1012	0.07	[0.06; 0.09]	5.4%	3.7%
Liu 2004	10	88	0.11	[0.06; 0.20]	0.7%	2.8%
Li 2011	7	116	0.06	[0.02; 0.12]	0.5%	2.6%
Wu 2008	18	177	0.10	[0.06; 0.16]	1.3%	3.2%
Xiao 2016	68	801	0.08	[0.07; 0.11]	4.9%	3.7%
Ding 2014	7	104	0.07	[0.03; 0.13]	0.5%	2.6%
Li 2008	7	93	0.08	[0.03; 0.15]	0.5%	2.6%
Liu 2014	25	46	0.54	[0.39; 0.69]	0.9%	3.0%
Ma 2014	33	130	0.25	[0.18; 0.34]	1.9%	3.4%
Wu 2008	19	274	0.07	[0.04; 0.11]	1.4%	3.3%
Liu 2014	0	5	0.00	[0.00; 0.52]	0.0%	0.5%
Wu 2009	5	71	0.07	[0.02; 0.16]	0.4%	2.3%
Liu 2013	4	61	0.07	[0.02; 0.16]	0.3%	2.0%
Zhao 2008	23	88	0.26	[0.17; 0.37]	1.3%	3.3%
Sun 2010	7	128	0.05	[0.02; 0.11]	0.5%	2.6%
Yang 2013	0	2	0.00	[0.00; 0.84]	0.0%	0.4%
Chao 2011	386	2104	0.18	[0.17; 0.20]	24.6%	3.9%
Lai 1999	8	81	0.10	[0.04; 0.19]	0.6%	2.7%
Ding 2008	27	227	0.12	[0.08; 0.17]	1.9%	3.4%
Chan 1996	2	7	0.29	[0.04; 0.71]	0.1%	1.2%
Chan 2012	105	418	0.25	[0.21; 0.30]	6.1%	3.8%
Lau 2015	51	236	0.22	[0.17; 0.27]	3.1%	3.6%
Liu 2008	13	86	0.15	[0.08; 0.24]	0.9%	3.0%
Hlaing 2010	6	35	0.17	[0.07; 0.34]	0.4%	2.3%
Lo 2002	51	680	0.08	[0.06; 0.10]	3.7%	3.7%
Chen 2009	59	644	0.09	[0.07; 0.12]	4.2%	3.7%
Wu 2006	3	152	0.02	[0.00; 0.06]	0.2%	1.8%
**Fixed effect model**		**12020**	**0.14**	**[0.13; 0.14]**	**100.0%**	--
**Random effects model**			**0.12**	**[0.10; 0.14]**	--	**100.0%**

Heterogeneity: $I^2 = 89\%$, $\tau^2 = 0.2904$, $p < 0.01$

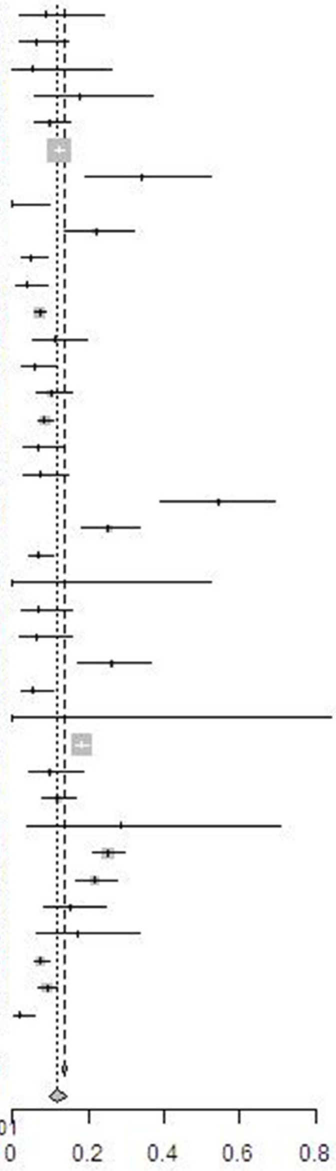

Supplementary Figure 2: Forest plot of the overall HPV18 prevalence in cervical cancer.

APPENDIX S1: SEARCH STRATEGIES FOR ELECTRONIC DATABASES

PubMed/MEDLINE (NCBI) 561 records returned on Nov.15, 2016

(Papillomaviridae[Mesh] OR Papillomavirus Vaccines[Mesh] OR Alphapapilloma*[tiab] OR Betapapilloma*[tiab] OR Gammapapilloma*[tiab] OR Mupapilloma*[tiab] OR papilloma*[tiab] OR HPV*[tiab]) AND (Cervical[tiab] OR cervix[tiab] OR uter*[tiab]) AND (Uterine Cervical Dysplasia[Mesh] OR Uterine Cervical Neoplasms[Mesh] OR HSIL*[tiab] OR high grade squamous intraepithelial[tiab] OR cancer*[tiab] OR tumor[tiab] OR tumors[tiab] OR tumoral*[tiab] OR neoplas*[tiab] OR tumour*[tiab] OR dysplasia*[tiab] OR dysplastic[tiab] OR carcino*[tiab] OR adenosquam*[tiab] OR adenocarcinoma*[tiab]) AND ((Female[Mesh] OR Female*[tiab] OR Women[Mesh] OR Woman[tiab] OR Women[tiab])) AND ((China[Mesh] OR Chinese[tiab] OR Taiwan[Mesh] OR Taiwanese[tiab])).

Cochrane central register of controlled trials (Wiley)

15 records returned on Nov.15, 2016 Last Saved: 15/11/2016 13:35:18.351; Description: ID Search #1: MeSH descriptor: [Papillomaviridae] explode all trees# 2: MeSH descriptor: [Papillomavirus Vaccines] explode all trees; #3: Alphapapilloma*; #4: Betapapilloma*; #5: Gammapapilloma*; #6: Mupapilloma*; #7: papilloma*; #8: HPV*; #9: #1 or #2 or #3 or #4 or #5 or #6 or #7 or #8; #10: Cervical; #11: cervix; #12: uter; #13: #10 or #11 or #12; #14: HSIL*; #15: high grade squamous intraepithelial; #16: cancer*; #17: tumors; #18: tumor; #19: tumoral*; #20: neoplas*; #21: tumour*; #22: dysplasia*; #23: dysplastic; #24: carcino*; #25: adenosquam*; #26: adenocarcinoma*; #27: MeSH descriptor: [Uterine Cervical Dysplasia] explode all trees; #28: MeSH descriptor: [Uterine Cervical Neoplasms] explode all trees; #29: #14 or #15 or #16 or #17 or #18 or #19 or #20 or #21 or #22 or #23 or #24 or #25 or #26 or #27 or #28; #30: MeSH descriptor: [Female] explode all trees; #31: MeSH descriptor: [Women] explode all trees; #32: Female*; #33: women; #34: woman; #35: #30 or #31 or #32 or #33 or #34; #36: Chinese; #37: MeSH descriptor: [China] explode all trees; #38: #36 or #37; #39: #9 and #13 and #29 and #35 and #38.

China national knowledge infrastructure(CNKI)

390 records returned on Nov.15, 2016; 条件： 题名=”HPV”+”人乳头瘤病毒”+”人乳头状瘤病毒” AND 关键词 = “宫颈病变”+”宫颈癌”+”宫颈上皮内瘤变”+”CIN” AND 题名 = “分型”+”亚型” and 发表时间 between 1979-01-01,2016-11-15 (精确匹配).

VIP database for chinese technical periodicals(VIP)

308 records returned on Nov.15, 2016; (中文题名=HPV+人乳头瘤病毒+人乳头状瘤病毒)*(中文关键词=宫颈病变+宫颈癌+宫颈上皮内瘤变+CIN)*(中文题名=分型+亚型.

Supplementary Table 1: Type-specific HPV positivity was shown stratified by cervical disease grade and geographical region. See_Supplementary_Table 1

Supplementary Table 2: Quality Assessment Tools Answers. See_Supplementary_Table 2

Supplementary Table 3: Type-specific HPV prevalence and prevalence ratios in 30165 HPV-positive women from China, stratified by grade of cervical disease. See_Supplementary_Table 3

Supplementary Table 4: Characteristics and type-specific prevalence of HPV DNA among Chinese female with cervical lesions, by study and region. See_Supplementary_Table 4

Supplementary Table 5: PRISMA 2009 Checklist. See_Supplementary_Table 5
